# Analysis of Net Primary Productivity Variation and Quantitative Assessment of Driving Forces—A Case Study of the Yangtze River Basin

**DOI:** 10.3390/plants12193412

**Published:** 2023-09-28

**Authors:** Chenxi Liu, Shuo Shi, Tong Wang, Wei Gong, Lu Xu, Zixi Shi, Jie Du, Fangfang Qu

**Affiliations:** 1 Electronic Information School, Wuhan University, Wuhan 430072, China; dawnlcx@whu.edu.cn (C.L.);; 2 State Key Laboratory of Information Engineering in Surveying Mapping and Remote Sensing, Wuhan 430079, China; 3 Perception and Effectiveness Assessment for Carbon-Neutrality Efforts, Engineering Research Center of Ministry of Education, Wuhan 430079, China; 4 Wuhan Institute of Quantum Technology, Wuhan 430206, China

**Keywords:** net primary productivity, climate change, human activities, residual analysis

## Abstract

Net primary productivity (NPP) can indirectly reflect vegetation’s capacity for CO${}_{2}$ fixation, but its spatiotemporal dynamics are subject to alterations to some extent due to the influences of climate change and human activities. In this study, NPP is used as an indicator to investigate vegetarian carbon ability changes in the vital ecosystems of the Yangtze River Basin (YRB) in China. We also explored the NPP responses to climate change and human activities. We conducted a comprehensive analysis of the temporal dynamics and spatial variations in NPP within the YRB ecosystems from 2003 to 2020. Furthermore, we employed residual analysis to quantitatively assess the contributions of climate factors and human activities to NPP changes. The research findings are as follows: (1) Over the 18-year period, the average NPP within the basin amounted to 543.95 gC/m${}^{2}$, displaying a noticeable fluctuating upward trend with a growth rate of approximately 3.1 gC/m${}^{2}$; (2) The areas exhibiting an increasing trend in NPP account for 82.55% of the total study area. Regions with relatively high stability in the basin covered 62.36% of the total area, while areas with low stability accounted for 2.22%, mainly situated in the Hengduan Mountains of the western Sichuan Plateau; (3) NPP improvement was jointly driven by human activities and climate change, with human activities contributing more significantly to NPP growth. Specifically, the contributions were 65.39% in total, with human activities contributing 59.28% and climate change contributing 40.01%. This study provides an objective assessment of the contributions of human activities and climate change to vegetation productivity, offering crucial insights for future ecosystem development and environmental planning.

## 1. Introduction

The increase in CO${}_{2}$ concentration will cause a series of problems such as global warming [1,2], changes in food production [3,4], and groundwater table drop [5]. Within terrestrial ecosystems, vegetation plays a crucial role in the carbon cycle by absorbing CO${}_{2}$ from the atmosphere through photosynthesis [6,7,8,9]. Hence, assessing the terrestrial ecosystems’ potential for carbon sequestration holds significant importance for governmental policy-making and ecological preservation.

Ecological systems sequester carbon through photosynthesis. The total amount of organic carbon accumulated by vegetation through photosynthesis in a unit of time is referred to as gross primary productivity (GPP) [10]. Deducting the portion consumed by vegetation through autotrophic respiration leaves the net primary productivity (NPP) [11]. NPP reflects the efficiency and intensity of carbon fixation within an ecosystem and is a critical indicator of ecosystem productivity [12], and is also more sensitive to factors such as climate and human interference [13]. Consequently, tracking the spatiotemporal dynamics of NPP over historical periods is imperative for forecasting future trends in carbon and ecosystem environmental changes.

The change in NPP is closely related to climate change [14,15]. The combined effects of climate change and other factors may cause irreversible impacts on the ecosystem [16], and moreover have more or lesser effects on vegetation carbon sequestration. Temperature, precipitation, and solar radiation are all key factors influencing vegetation growth. Warming temperatures lead to changes in vegetation phenology, with an earlier onset of spring phenology and a delayed onset of autumn phenology [17,18,19]. Additionally, the temperature increase leads to a reduction in the temperature difference between summer and winter, impacting vegetation photosynthesis and altering vegetation productivity [20]. In tropical regions, studies have shown that intra-annual temperature variations lead to more pronounced vegetation periodicity, with temperature changes having a greater impact compared to other climatic variables [21]. The availability of water for vegetation is the foremost factor in ensuring vegetation growth and the degree of vegetation dependence on available water varies significantly [22]. The sensitivity of vegetation productivity to precipitation varies across different regions, with arid regions being more sensitive compared to humid areas, and grasslands and shrublands exhibiting higher sensitivity compared to forests and wetlands [23]. Similarly, extreme rainfall events can increase vegetation net productivity in arid regions while having a negative impact on vegetation in humid areas [24]. Solar radiation is the essential energy source for vegetation growth, and a decrease in solar radiation within a certain range will result in a decline in vegetation productivity [25].

The YRB, Asia’s largest river, traverses the vast inland regions of China, encompassing significant wetlands, extensive grasslands, and diverse forest ecosystems. Notable wetlands in the area include the Poyang Lake Wetland, Dongting Lake Wetland, and the Three Gorges Reservoir Wetland. These wetland areas [26] play pivotal roles in ecological equilibrium, serving as crucial habitats for rare wildlife and exerting essential influence on water resource conservation and regulation. Grasslands [27] provide a vital source of sustenance for livestock, contributing significantly to soil erosion mitigation, the promotion of biodiversity, and the preservation of ecological balance. Forests [28] blanket the mountainous and hilly terrains throughout the YRB, fulfilling critical ecological functions such as oxygen generation, soil and water conservation, and carbon cycling. It is not only a valuable ecological barrier [29,30,31], but also a significant carbon pool in China’s terrestrial ecosystem. Predecessors have carried out a significant amount of research in the YRB, mainly focusing on the vegetation index [32,33,34], but there is still a lack of in-depth understanding of the vegetation productivity and its driving mechanisms. Yang et al. [35] conducted research on the impact of NPP from the perspective of land use change. Wang et al. [36] explored the increasing trend and influencing factors of NPP between 2000 and 2014, and the results indicated that human activities were the primary driver of NPP changes. Zhang et al. [37] used the LPJ (Lund–Potsdam–Jena) model and NDVI vegetation index to simulate NPP in the YRB from 1982 to 2013. Hence, there remains a deficiency in research that adequately distinguishes the impacts of climate change from the interferences caused by human activities and quantifies the relative contributions of human activities and climate change.

With the development of afforestation projects, the dynamic changes in NPP spatial distribution, and the response of NPP to climate and human-induced interventions, there is a need for further exploration. Therefore, this research mainly includes the following points: (1) Describing the spatiotemporal dynamics of NPP in the YRB; (2) Investigating the relationship between NPP and climate factors as well as human-induced interventions, and quantitatively characterizing their contributions to NPP. It is expected that this research will provide an essential scientific basis for an in-depth understanding of the long-term carbon dynamic change mechanism in the YRB and for future scientific ecosystem monitoring and assessment.

## 2. Data and Methods

### 2.1. Study Area

**Figure 1:**
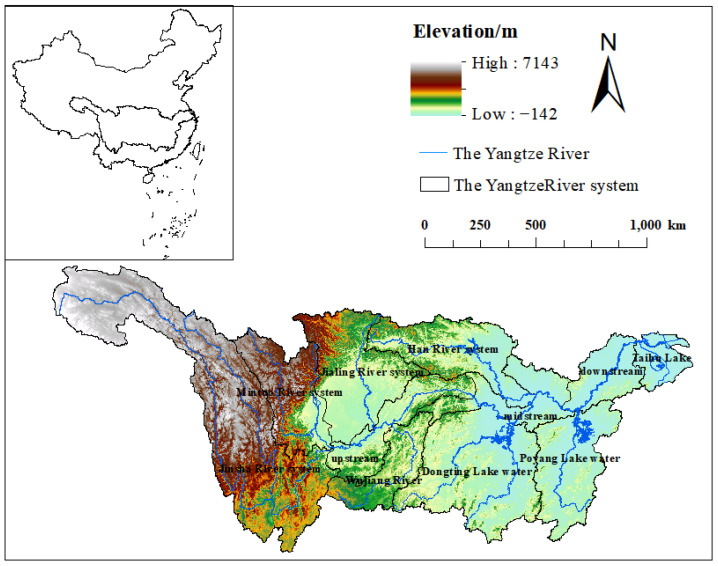
The Yangtze River Basin.

The YRB (Figure 1) is the largest river in Asia, originating from the Qinghai-Tibet Plateau. The YRB covers an area of $1.8\times 10^{7}$ km${}^{2}$ and occupies about 18.8% of China’s land area. In this study, the YRB is divided into 11 water systems, including Poyang Lake, Dongting Lake, Mintuo River, Jinsha River, Wujiang River, Han River, Taihu Lake, Jialing River water system, upstream, middle, and downstream.

The Yangtze River flows through plateaus, mountains, basins, plains, hills, etc., showing a multi-level stepped terrain and complex terrain. It belongs to the subtropical monsoon climate. The annual precipitation in the basin is uneven, and the interannual variation of heavy rainfall is significant. According to statistics, the YRB has $3237.9\times 10^{9}$ m${}^{3}$ of forest resources, equivalent to a quarter of the country’s total stock volume. The forest areas are mainly located in northern Yunnan, western Sichuan, western Hunan, western Hubei, and Jiangxi, and the area of the economic forests ranks first in the country.

### 2.2. Data Source

The research data are obtained through the Google Earth Engine (GEE) platform https://earthengine.google.com/, accessed on 6 September 2022), which can provide satellite data products on a global scale. The GEE’s powerful computing power is used to solve research questions on spatiotemporal large scales [38,39,40]. The research data mainly include NPP (https://developers.google.com/earth-engine/datasets/catalog/MODIS_061_MYD17A3HGF/, accessed on 6 September 2022), meteorological data (temperature, solar radiation precipitation, https://developers.google.com/earth-engine/datasets/catalog/ECMWF_ERA5_LAND_HOURLY/ (accessed on 21 October 2022), https://developers.google.com/earth-engine/datasets/catalog/UCSB-CHG_CHIRPS_DAILY#bands/ (accessed on 30 October 2022)). We acquire NPP data through the MYD17A3HGF V6 product, with a spatial resolution of 500 m and a temporal resolution of 8 days. The maximum value composites (MVCs) technique derives annual maximum NPP data. Precipitation data are from CHIRPS Daily Version 2.0 products, with a temporal resolution of daily and a spatial resolution of 0.05°, capturing annual total precipitation. Temperature and solar radiation data are from ERA5 Land Hourly data, with a spatial resolution of 0.1°. We also calculate annual mean temperature values and annual total solar radiation. The digital elevation model (DEM) is derived from the SRTM digital elevation image product https://srtm.csi.cgiar.org/srtmdata/ (accessed on 6 September 2022) with a spatial resolution of 30 m.

### 2.3. Methods

**Figure 2:**
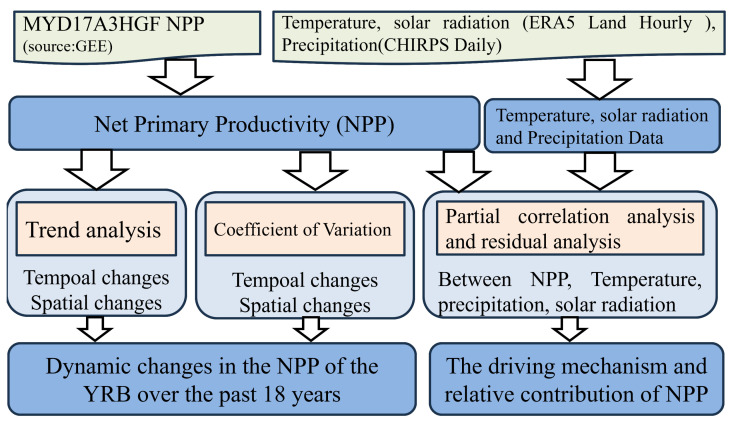
The technical approach of the research.

Figure 2 represents our main research process. We obtain the NPP data through the MYD17A3HGF product. On this basis, we obtain 18 years of NPP data and analyze the spatiotemporal changes of NPP using the Theil-Sen method. The CV index provides insight into the stability of NPP changes over the 18-year period. We further analyze the correlation between NPP and climate factors using the partial correlation analysis method. The land cover changes in 2003, 2005, 2010, 2015, and 2020 are observed through the land use transfer matrix, allowing us to assess the impact of human activities on NPP resulting from land cover changes.

#### 2.3.1. Trend Analysis Method

The Theil-Sen method [41] analyzed the interannual dynamic changes of NPP from 2003 to 2020.

(1) $$\beta =\mathrm{Median}\left(\frac{x_{k}-x_{i}}{k-i}\right),\forall k>i$$

where $x_{k}$, $x_{i}$ is the value of NPP at *k*, *I*, where 1<i<k<n. When β>0, it means an increase; otherwise, it means a decrease.

Mann–Kendall is a non-parametric statistical test method [42]. Its advantage is that it does not require the trend to be linear and is not affected by outliers. The formula is

(2) $$Z_{C}=\left\{\begin{array}{c} S-\frac{1}{\sqrt{\mathrm{var}(S)}},S>0\\ 0,\;S=0\\ \;S+\frac{1}{\sqrt{\mathrm{var}(S)}},S<0 \end{array}\right.$$

(3) Var(S)=n(n−1)(2∗n+5)/18

(4) $$S=\sum_{i=1}^{n-1}\sum_{k=i+1}^{n}\mathrm{sgn}\left(x_{k}-x_{i}\right)$$

(5) $$\mathrm{sgn}\left(x_{j}-x_{i}\right)=\left\{\begin{array}{c} 1,x_{j}-x_{i}>0\\ 0,x_{j}-x_{i}=0\\ -1,x_{j}-x_{i}<0 \end{array}\right.$$

where *n* is the count of the sample. In this paper, give *n* = 18, and the relationship between the Z value and the reliability is shown in the Table 1.

#### 2.3.2. Stability Analysis

The coefficient of variation (CV) [43] is used to reflect the fluctuation degree of *NPP* from 2003 to 2020. The stability distribution of NPP is obtained by calculating the *CV* value of NPP at each pixel during the study period. It is calculated as follows:

(6) $$CV=\frac{1}{\bar{NPP}}\sqrt{\frac{\sum_{i=1}^{n}{\left(NPP_{i}-\bar{NPP}\right)}^{2}}{n}}$$

where $NPP_{i}$ is the NPP value in the *i*-th year, *n* represents the total number of years, and $\bar{NPP}$ is the annual average NPP.

#### 2.3.3. Partial Correlation Analysis

Partial correlation analysis is used to analyze the linear correlation between two variables while controlling for the linear effects of other variables [44]. The judgment index is the *R* of the correlation coefficient value.

(7) $$P_{ij,k}=\frac{P_{ij}-P_{ik}P_{jk}}{\sqrt{\left(1-P_{ik}^{2}\right)\left(1-P_{jk}^{2}\right)}}$$

Suppose there are 3 variables *i*, *j*, and *k*, while $P_{ij,k}$ and *k* are partial correlation coefficients between independent variable *k* and independent variables *i* and *j*; $P_{ij}$, $P_{ik}$, $P_{jk}$ are correlation coefficients between two factors. According to *R* and *P*, we obtain three different significance levels: significant positive correlation (R>0, P<0.05), no significant correlation (P≥0.05), and significant negative correlation (R<0, P<0.05).

#### 2.3.4. Multivariate Regression Residual Analysis

To quantitatively separate the effects of climate change and human activities on NPP variation, this study defined three categories of NPP: a multivariate regression model was constructed using solar radiation, temperature, and precipitation data to derive NPP predictions (PNPP). NPP values obtained from remote sensing imagery represented actual values (ANPP). NPP residual values (HNPP), which represent the impact of human activities on NPP, were calculated as the difference between ANPP and PNPP. When HNPP is greater than zero, it indicates a promotional effect of human activities on NPP, while values less than zero indicate inhibition.

(8) HNPP=ANPP−PNPP

(9) PNPP=a×P+b×T+c×S+d

where P represents annual precipitation, T represents annual mean temperature, S represents annual total solar radiation; a, b, and c are regression coefficients; and d is the constant term.

Linear regression is employed to calculate the change slopes for the three NPP types, elucidating vegetation growth factors and quantifying their relative contribution rates (Table 2).

## 3. Results

### 3.1. Spatial Distribution of Average NPP

**Figure 3:**
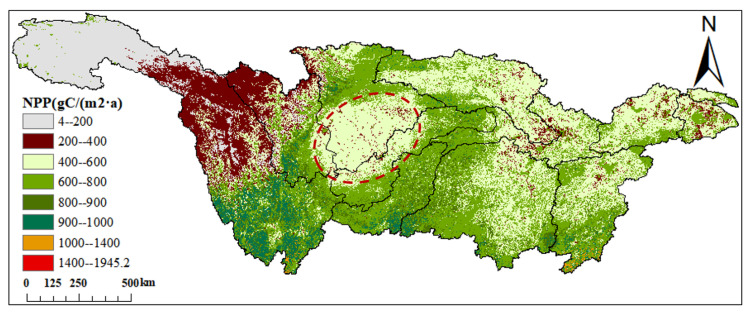
Spatial distribution of average NPP from 2003 to 2020 (the red circle in the figure is the Sichuan Basin, and the black area is the area without data).

From the spatial perspective (Figure 3), NPP exhibits distinct regional characteristics, with higher values in the southern and eastern parts compared to the northern and western regions. The multi-year average NPP is 543.95 gC/(m${}^{2}\cdot a$). Areas with an average NPP ranging from 0 to 400 gC/(m${}^{2}\cdot a$) account for 22.56% of the entire watershed, primarily distributed in the northwestern parts of the Jinsha River and Mintuo River watersheds. Regions with NPP ranging from 400 to 600 gC/(m${}^{2}\cdot a$) cover approximately 35.72% of the area and are found in the Sichuan Basin, Jianghan River Basin, middle and lower reaches of the YRB, Taihu Lake, and Poyang Lake areas to the north. Areas with NPP ranging from 600 to 800 gC/(m${}^{2}\cdot a$) account for about 30.20% and are predominantly distributed in the vicinity of the Sichuan Basin, Dongting Lake watershed, and the southern part of the Poyang Lake watershed. Regions with NPP ranging from 800 to 1000 gC/(m${}^{2}\cdot a$) make up approximately 10.82% of the area, predominantly situated in the southern part of the Jinsha River watershed. NPP values ranging from 1000 to 1945.2 gC/(m${}^{2}\cdot a$) account for only 0.26% of the entire study area. The Sichuan Basin features a closed topography, a higher latitude resulting in relatively higher temperatures, and abundant precipitation, but lower solar radiation compared to other latitudes. In summary, these unique climatic factors contribute to slightly lower NPP values in this region compared to the surrounding areas.

### 3.2. Time-Series Characteristics of NPP

**Figure 4:**
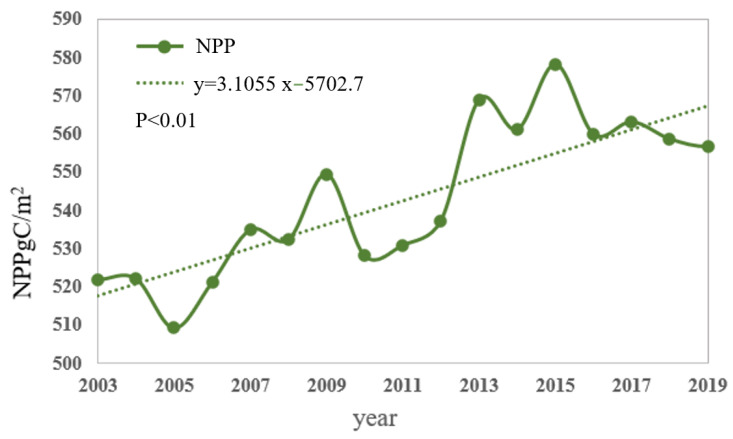
Annual mean values of NPP in the YRB.

From 2003 to 2020, the NPP in the YRB exhibited a noticeable fluctuating upward trend, with an approximate growth rate of 3.1 gC/(m${}^{2}\cdot a$) (Figure 4). In 2010, there was a significant decrease in NPP, followed by a substantial increase in 2013. The data indicate that, in July 2010, the YRB was severely affected by heavy rainfall, leading to a catastrophic rainstorm disaster that caused extensive damage to vegetation and farmland. The annual average NPP in 2003 and 2020 was 521.77 gC/(m${}^{2}\cdot a$) and 560.29 gC/(m${}^{2}\cdot a$), respectively. The maximum NPP was recorded in 2015, while the minimum was observed in 2005, with a difference of 68.92 gC/(m${}^{2}\cdot a$).

**Figure 5:**
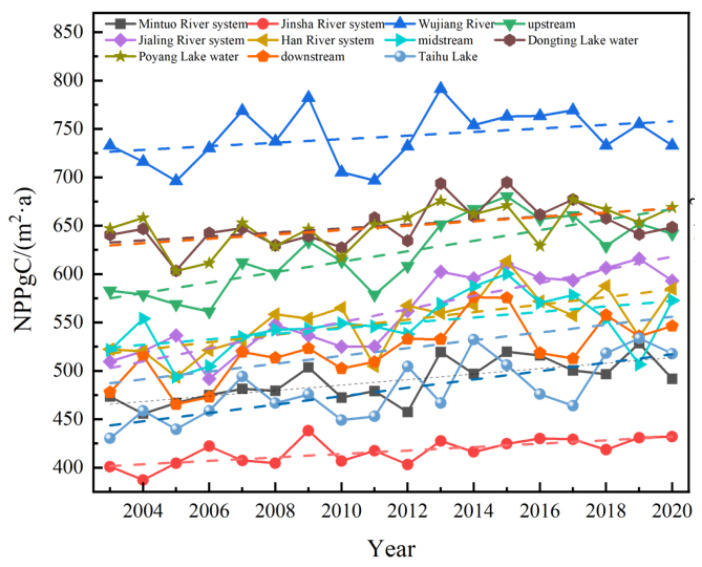
The annual mean value of NPP of each water system in the YRB.

In the 11 river systems within the YRB (Figure 5), NPP exhibited varying degrees of growth. The Wujiang had the highest NPP value, with annual averages of 742.2 gC/(m${}^{2}\cdot a)$. The Jinsha River basin had the lowest NPP value, with an annual average of 416.7 gC/(m${}^{2}\cdot a)$.

Specifically, the upper and Jialingjiang River NPP growth rates were 5.42 gC/(m${}^{2}\cdot a)$ and 6.77 gC/(m${}^{2}\cdot a)$, respectively, while the Jinsha River and Wujiang River NPP growth rates were 1.77 gC/(m${}^{2}\cdot a)$ and 1.83 gC/(m${}^{2}\cdot a)$, respectively. Regions with slower NPP growth included the Mintuo, Dongting Lake, Poyang Lake, and the middle reaches, with growth rates of 2.84, 2.06, 2.25, and 2.83 gC/(m${}^{2}\cdot a)$, respectively. Growth rates in other river systems ranged from 3.9 gC/(m${}^{2}\cdot a)$ to 4.32 gC/(m${}^{2}\cdot a)$.

### 3.3. Spatial Distribution of Change Trends of NPP

**Figure 6:**
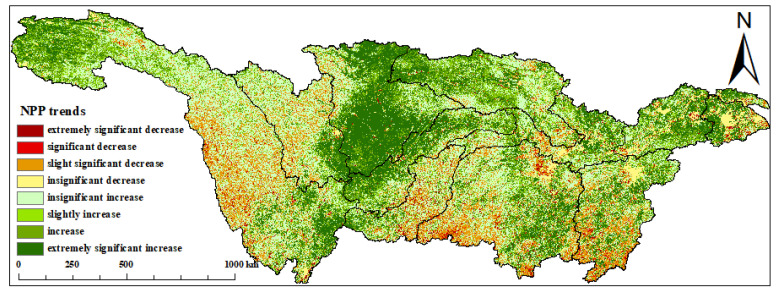
The 2003–2020 spatial change trend of NPP.

From 2003 to 2020, the areas where the NPP is growing accounted for 82.55% of the whole research area (Figure 6). This shows that the carbon sequestration capacity of the ecosystem in the YRB has increased.

Regions with a significant increase in NPP (including highly significant and significant increases) account for 37.87% of the entire basin. These areas are mainly located in the upper reaches of the Jialingjiang River system and are also scattered in the Hanshui River system, the middle reaches, the lower reaches, the Taihu Lake, and the upper reaches of the Jialingjiang River. In recent years, China has implemented comprehensive protection and rehabilitation of the ecological setting in the headwaters of the YRB, strengthened the management of degraded grasslands, and continuously improved the ecosystem’s functionality [45]. Regions with a significant decrease in NPP (including highly significant and significant decreases) account for only 3.03% of the entire study area, primarily concentrated in Dongting Lake, south of the Wujiang River, and the northeastern part of Dongting Lake. Stable NPP regions account for 37.89% of the study area.

**Figure 7:**
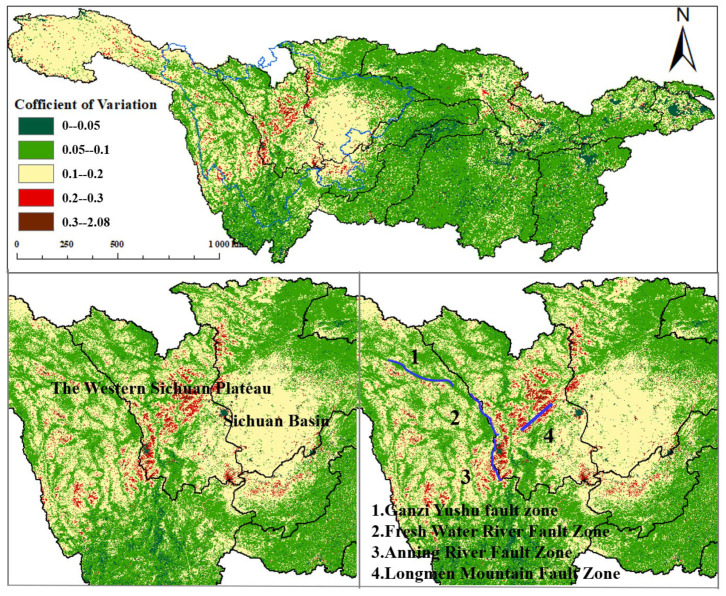
The spatial change trend of NPP from 2003 to 2020.

The results of the stability analysis (Table 3 and Figure 7) show that the coefficient of variation within the YRB has obvious spatial differences over the past 18 years, and the overall fluctuation is relatively low, accounting for 62.36%. In the research area, stability, lower stability, and low stability accounted for 35.42%, 1.93%, and 0.29%, respectively. Among them, in the Sichuan Basin, the upper part of the Jinsha River system is dominated by medium stability, and its lower stability is scattered among them. The stability of the central part of the Mintuo River system and the Jinsha River system is relatively low, which is mainly caused by multiple earthquakes in the structural fault zone. The landslides caused by the earthquake caused extensive destruction of vegetation, which further caused NPP fluctuation [46].

### 3.4. Correlation Analysis between Climate Change and NPP Variation

#### 3.4.1. Spatiotemporal Characteristics of Climate Factors

**Figure 8:**
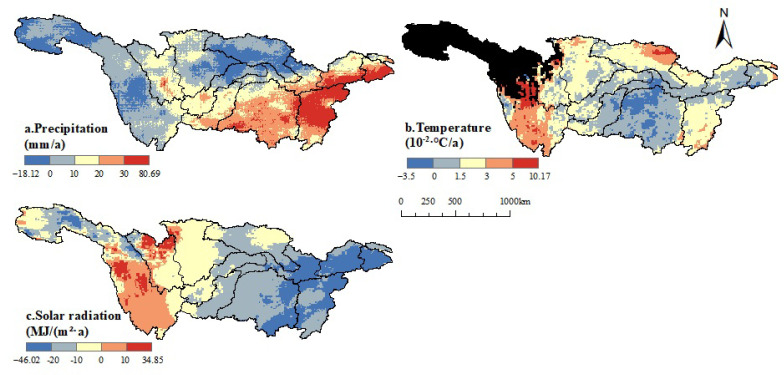
Spatial distribution of climate factor trends from 2003 to 2020.

As shown in the Figure 8, the climate in the YRB has undergone a warming and humidifying trend, and there are notable spatial variations in climate factor changes. The fluctuation range of precipitation is from −18.12 to 80.69 mm/a, with a mean value of 11.88 mm/a. In the study area, 81.98% of the regions exhibit an increasing trend in precipitation, while 18.02% show a decreasing trend. The areas with increasing trends are mainly located in the southern part of the Hanjiang River, the northern part of the upper reaches, the eastern part of the Jialing River, and the northwestern and central parts of the Minjiang River.

Temperature primarily shows an increasing trend, with a fluctuation range from −0.035 to 0.1017 °C/a and a mean of 0.0178 °C/a. The regions with a decreasing temperature trend are mainly found in the northwestern part of the Dongting Lake Basin, accounting for 6.74% of the area. Solar radiation has a fluctuation range from −46.02 to 34.85 MJ/(m${}^{2}\cdot a)$, with a mean of −10.41 MJ/(m${}^{2}\cdot a)$. The majority of the region experiences a decreasing trend in solar radiation, covering 83.15% of the area. The areas with increasing trends account for 16.85% and are mainly distributed in the northern part of the Minjiang River and the southern part of the Jinsha River.

#### 3.4.2. Partial Correlation Analysis between NPP and Climate Factors

**Figure 9:**
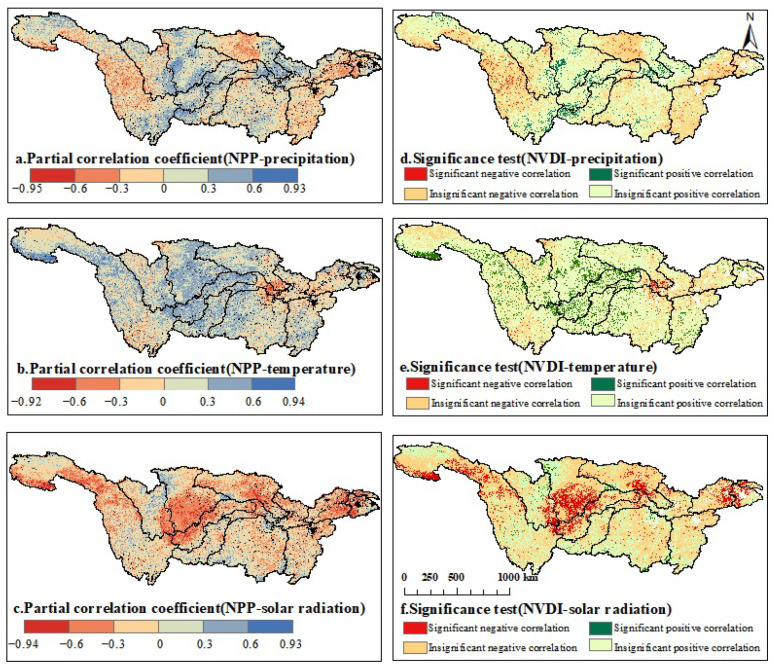
The 2003–2020 partial correlation coefficient between NPP and temperature.

Partial correlation and T-test were conducted to analyze the pixel-wise correlations between NPP and time-series data of temperature, precipitation, and solar radiation from 2003 to 2020 (Figure 9). The range of partial correlation coefficients between temperature and NPP was from −0.92 to 0.94, with 76.20% of the regions showing a positive correlation. Among them, 9.54% of the regions exhibited a significant positive correlation, mainly scattered in the upper reaches, Wujiang, the eastern part of Jialingjiang, the western part of Dongting Lake, and the western part of the middle reaches. The negative correlation was observed in 23.80% of the regions, with 0.98% exhibiting a significant negative correlation, mainly distributed in the middle part of the middle reaches and the southern part of Hanjiang.

The partial correlation coefficients between precipitation and NPP ranged from −0.95 to 0.93, with 58.44% of the regions showing a positive correlation. Among them, 4.68% exhibited a significant positive correlation, mainly concentrated in the western and southern parts of Wujiang, the upper and middle parts of the middle reaches, and the southeast part of Hanjiang. The negative correlation was observed in 41.57% of the regions, with 2.08% exhibiting a significant negative correlation, mainly scattered in the middle parts of Jialingjiang and Hanjiang.

Solar radiation had partial correlation coefficients ranging from −0.94 to 0.93 with NPP. A total of 31.93% of the regions showed a positive correlation, with 1.61% exhibiting a significant positive correlation, scattered in the northwestern part of Jialingjiang and at the junction of Jialingjiang and Hanjiang. A total of 68.07% of the regions exhibited a negative correlation, with 10.89% showing a significant negative correlation, mainly concentrated in the Sichuan Basin, the junction of Taihu Lake and the lower reaches, the middle and southwestern parts of Jialingjiang, and the middle part of Hanjiang.

### 3.5. Driving Mechanisms and Relative Contributions to NPP Variability

#### 3.5.1. Driving Mechanisms of NPP Variability

**Figure 10:**
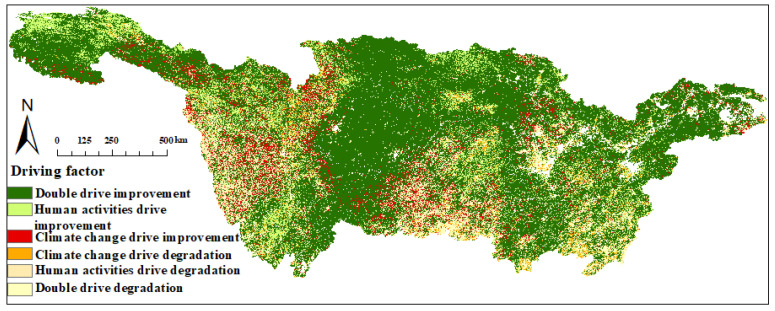
Spatial distribution of NPP change drivers.

The area within the basin where NPP has improved due to the joint influence of human activities and climate change accounts for 65.39% (Figure 10). It is primarily located in the Jialing River Basin, upper reaches, Hanjiang River Basin, and downstream regions. The area solely improved by human activities accounts for 10.42%, mainly scattered in the southern parts of the Jialing River Basin, southern parts of the Hanjiang River Basin, southwestern parts of the Hanjiang River Basin, and the junction of the Jialing River Basin. The area solely improved by climate change accounts for 6.31%, with a relatively scattered distribution, scattered in the central parts of the Jinsha River Basin and the central parts of the Min River Basin. The area degraded due to the joint influence of human activities and climate change accounts for 8.76%, with similarly scattered distribution, mainly scattered in the southern parts of the Poyang Lake Basin, southeastern parts of the Dongting Lake Basin, and the central parts of the Wujiang River and Jinsha River Basin. The regions degraded solely by human activities and solely by climate change account for 5.15% and 3.37%, respectively. Overall, it can be observed that, under the joint influence of climate and humans, NPP exhibits an overall positive development trend.

#### 3.5.2. Relative Contributions of Different Influential Factors to NPP Variations

**Figure 11:**
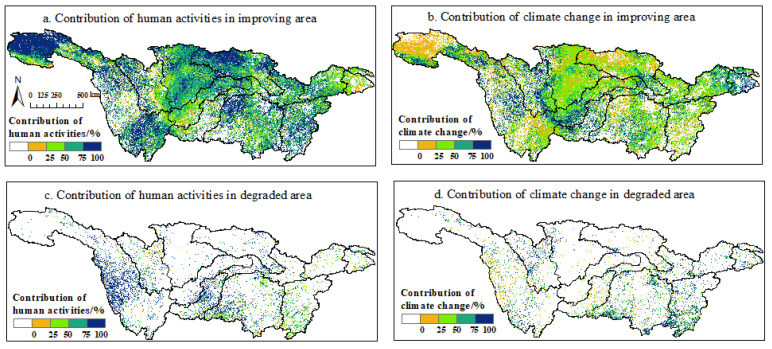
Contribution rates of different influencing factors to NPP variations.

In this section, the relative contributions of human activities and climate change to NPP changes in the YRB are calculated based on the slopes of NPP, HNPP, and PNPP variations (Figure 11). Overall, human-induced interventions contribute 59.28% to the total, while climate change contributes 40.01%. Further analysis of the NPP improvement areas shows that the relative contributions of human-induced interventions and climate variability are 59.95% and 39.70%, respectively. In the NPP degradation areas, the relative contributions of human-induced interventions and climate variability account for 56.12% and 41.46%, respectively. In the entire study area, human intervention predominates, accounting for 4.69% of the NPP improvement areas. Climate change, on the other hand, dominates, constituting 2.83% of the NPP improvement areas. Regions where human activities contribute more than 50% to NPP account for 53.76% and are mainly located in the northwest of the Jinsha River Basin, the western part of the Hanjiang River Basin, and the eastern part of the Jialing River Basin. Regions where climate change contributes more than 50% to NPP account for 25.27% and are scattered across various river basins.

## 4. Discussion

### 4.1. Spatiotemporal Distribution and Change of NPP

The YRB is rich in natural resources. Against the backdrop of climate change, efforts have been made in ecological and environmental management in the YRB, yielding certain achievements [47,48,49]. From 2003 to 2020, there was an overall trend of fluctuating increase in NPP, and the NPP for each river system also exhibited an upward trend.

Previous research has demonstrated a strong correlation between estimated NPP values and vegetation NDVI [50,51,52,53], with NPP increasing as vegetation quality improves. The NPP in the Sichuan Basin, the middle reaches of Dongting Lake, and the upper reaches of the Jinsha River have exhibited varying degrees of growth. The Sichuan Basin, in particular, has shown a notable increase, with a predominance of moderate stability, along with scattered areas of lower stability [54,55]. This suggests that the reforestation efforts by the Sichuan municipal government in recent years have been effectively executed. The overall NPP in the Han River Basin is also exhibiting an increasing trend, especially in regions such as Hanzhong and Shiyan, aligning with the findings of Zhang et al.’s research [56]. The source area of the YRB is a typical high-altitude and cold plateau region, with an ecosystem highly sensitive and fragile, making its carbon balance particularly responsive to climate and anthropogenic factors. In recent years, there has been a significant increase in NPP in the source area, as indicated in Figure 11, with a substantial contribution from anthropogenic disturbances [57], accounting from 75% to 100%. The Chinese government’s efforts in environmental protection in this context should not be underestimated.

Looking at the fluctuations in NPP over the years, there are significant high-variance regions in the Mintuo River. The central region of the Jinsha River Basin (Sichuan Province) also shows pronounced interannual variations. These areas are primarily located near the boundaries of China’s first and second terraces in the vicinity of the Hengduan Mountains (Ranges), characterized by mountainous terrain and tectonic fault zones [58]. Taking the Longmenshan seismic belt as an example, this belt has experienced numerous earthquakes. Frequent earthquakes not only cause damage to local buildings and structures but also trigger events like landslides, mudslides, and avalanches [59], resulting in significant harm to vegetation and its growth environment. In summary, geological disasters lead to a decline in NPP, while post-disaster reconstruction and natural recovery increase vegetation coverage and NPP. However, the instability of fault zones contributes to NPP fluctuations [60,61,62,63].

### 4.2. Relationship between Affecting Factors and NPP

The carbon sequestration of ecosystems is intricately linked to vegetation, and the growth of vegetation is influenced by diverse factors, including anthropogenic actions and climate variability. Climate change contributes significantly to the variation in NPP, accounting for 40.01% of the relative contribution and thus serving as a crucial factor affecting vegetation productivity changes. In the period from 2003 to 2020, the YRB experienced a warming and humidifying trend. The effects of temperature, precipitation, and solar radiation on vegetation NPP differ noticeably. Temperature and precipitation are positively correlated with NPP, accounting for 76.20% and 58.44% of the regions, respectively, while solar radiation is negatively correlated, covering 67.07% of the regions. However, the overall areas showing significant correlations are relatively small, considering that this is due to not accounting for the lag effects of vegetation on climate [64].

Solar radiation is a necessary condition for photosynthesis in vegetation, and solar radiation is directly proportional to the altitude. Due to its lower altitude, the Sichuan Basin consistently experiences lower solar radiation throughout the year compared to other regions [65]. In this region, solar radiation is significantly negatively correlated with NPP. Ge reached the same conclusion [66]. Additionally, when compared to other regions at the same latitude, the vegetation NPP in this area is noticeably lower.

The precipitation in the YRB increases from west to east, with a positive correlation area of 58.44%. However, the significantly positively correlated area is only 4.68%. This is due to the uneven distribution of annual precipitation in the YRB, where frequent heavy rainfall events occur, but the area and intensity of these heavy rainfall events vary greatly. Previous studies have mentioned [67] that, from 2016 to 2020, the YRB experienced different numbers, intensities, and distribution areas of heavy rainfall events in June and July, with rainfall events numbering 7, 4, 8, 9, and 9, respectively.

In general, the temperature in the YRB basin gradually decreases from east to west. The Jinsha River and Minto River basins, starting from the northwest of the Hengduan Mountains, exhibit more pronounced NPP stage changes. The NPP values vary from 200 to 400 gC/m${}^{2}$ in the area from the Hengduan Mountains to the source of the YRB, while the NPP variation in the source of the YRB ranges from 0 to 200 gC/m${}^{2}$. The average annual temperature at the source of the YRB is below 0 °C [68], and excessively low temperatures often suppress the vegetation’s growth, leading to reduced vegetation productivity.

The relative contribution of human activity change is 59.28%, serving as the primary driver for the increase in NPP productivity in the YRB. Starting in 2003, the implementation of the "Grain for Green Program" marked a historic transition from deforestation and land cultivation toward afforestation and reforestation. The YRB played a pivotal role in this transformation, serving as the primary battleground for reforestation efforts. Over the past two decades, the Chinese government has implemented the “Grain for Green Program”, covering more than 80 million mu (about 5.33 million hectares) in the YRB, with a yearly average reduction of 3.45% in land desertification, primarily in the southwestern regions. Compared to the urbanization process, the “Grain for Green Program” has played a more significant role in promoting the increase in NPP in forested areas [69]. Although urbanization has led to a continuous expansion of urban areas, there has been a greening trend in some core urban areas’ surroundings [70]. Similar conclusions have been drawn by researchers such as Peng [71] and Yang [72] for regions where the NPP has shown a significant increase, such as the YRB source area, Sichuan Basin, and the upper reaches of the Han River.

### 4.3. Limitations and Recommendations

NPP serves as an important indicator for assessing the capacity of vegetation to sequester atmospheric CO${}_{2}$ and evaluating ecosystem functionality. With the ongoing changes in global climate, future research necessitates a more in-depth exploration of the impact of climatic factors on the long-term variations in NPP. On the basis of enhancing data spatial resolution, it is essential to broaden the research’s temporal scale, for example, by investigating variations in NPP across monthly and quarterly time scales for different vegetation types [73]. Additionally, it would be beneficial to conduct hysteresis analysis [74] of various vegetation types’ NPP responses to climate change, aiming to better comprehend the challenges posed by climate change to ecosystems. Concurrently, human activities, in accordance with local climatic conditions, should engage in rational practices such as agriculture and the development of forest resources.

## 5. Conclusions

From 2003 to 2020, the annual average NPP in the YRB, despite fluctuations, showed an overall increasing trend with a growth rate of 3.1 gC/(m${}^{2}\cdot a)$. All 11 watersheds within the basin exhibited fluctuating upward trends, with the Middle Reaches and the Wujiang watershed showing larger fluctuations and growth rates of 1.8 gC/(m${}^{2}\cdot a)$ and 2.8 gC/(m${}^{2}\cdot a)$, respectively. Regions with significant NPP growth accounted for 37.87% of the total area, with the Jialing River watershed having the largest increase in NPP. NPP low-stability regions within the YRB from 2003 to 2020 covered 2.22% and were primarily located in the Hengduan Mountains of the western Sichuan Plateau.

During the period from 2003 to 2020, against the backdrop of a warming and humid climate in the YRB, NPP exhibited varying responses to different influencing factors. Overall, NPP showed a positive correlation with temperature and precipitation but a negative correlation with solar radiation. Temperature had a more significant impact on vegetation productivity than solar radiation and precipitation, making it the primary climatic factor influencing NPP variations.

Climate variability and anthropogenic actions exert a dual impact on NPP, with 74.15% of the areas being affected by the combined influence of climate variability and anthropogenic actions. Overall, NPP in the basin primarily shows improvement. The areas dominated by human activities in NPP improvement account for 10.42%, while those dominated by climate change contribute 6.3% to the improvement. Additionally, human activities emerge as the primary driving factor for NPP changes, with human activities and climate change contributing 59.28% and 40.01%, respectively.

## Figures and Tables

**Table 1:** The reliability table.

Absolute Value of Z	Reliability
≥1.65	90%
≥1.96	95%
≥1.96	99%

**Table 2:** Contribution rate calculation under different driving factors conditions.

$\theta_{\mathrm{ANPP}}$	$\theta_{\mathrm{HNPP}}$	$\theta_{\mathrm{PNPP}}$	Driving Factor	Relative Contribution/%	
				Climate Change	Human Activities
	>0	>0	Double-driven improvement	$\frac{\theta_{HNPP}}{\theta_{HNPP}+\theta_{ANPP}}$	$\frac{\theta_{ANPP}}{\theta_{HNPP}+\theta_{ANPP}}$
>0	>0	<0	human-driven improvement	100	0
	<0	>0	climate-driven improvement	0	100
	<0	<0	Double-driven degradation	$\frac{\theta_{HNPP}}{\theta_{HNPP}+\theta_{ANPP}}$	$\frac{\theta_{ANPP}}{\theta_{HNPP}+\theta_{ANPP}}$
<0	<0	>0	human-driven degradation	100	0
	>0	<0	climate-driven degradation	0	100

**Table 3:** Statistical table of NPP stability changes in the YRB.

Coefficient of Variation	Stability	Area Ratio
[0,0.05)	High stability, low volatility	6.99%
[0.05,0.1)	Higher stability, relatively low volatility	55.37%
[0.1,0.2)	Medium stability	35.42%
[0.2,0.3)	lower stability, relatively high volatility	1.92%
[0.3,+∞)	low stability, high volatility	0.29%

## Abbreviations

The following abbreviations are used in this manuscript:

YRB	Yangtze River Basin
NPP	Net primary productivity
GPP	Gross primary productivity
GEE	Google Earth Engine
